# Effects of different growth temperatures on growth, development, and plastid pigments metabolism of tobacco (*Nicotiana tabacum* L.) plants

**DOI:** 10.1186/s40529-018-0221-2

**Published:** 2018-02-05

**Authors:** Li Yun Yang, Shuang Long Yang, Jun Ying Li, Jun Hong Ma, Tao Pang, Cong Ming Zou, Bin He, Ming Gong

**Affiliations:** 1 School of Life Sciences, Engineering Research Center of Sustainable Development and Utilization of Biomass Energy, Ministry of Education, Key Laboratory of Biomass Energy and Environmental Biotechnology of Yunnan Province, Yunnan Normal University, Kunming, 650500 People’s Republic of China; 2 Yunnan Academy of Tobacco Agricultural Sciences, Kunming, 650031 People’s Republic of China; 3 Yunnan Tobacco Leaf Company, Kunming, 650218 People’s Republic of China

**Keywords:** Growth temperature, Tobacco plants, Growth and development, Plastid pigments metabolism, Regulation

## Abstract

**Background:**

Temperature remarkably affects the growth and metabolism of plants. Tobacco is an important cash crop, and the long-term effects of different growth temperatures (18.5, 23.5 and 28.5 °C, daily average) on growth, development and plastid pigments metabolism of tobacco plants were investigated in this study.

**Results:**

Compared with tobacco plants grown under 23.5 °C, treatments with 18.5 and 28.5 °C inhibited the expansion of leaves. The contents of superoxide anion (O_2_^·−^), hydrogen peroxide (H_2_O_2_) and malonaldehyde (MDA) in the leaves were significantly increased under 28.5 °C from 0 to 60 days, which in turn accelerated the flowering and senescence of tobacco plants. By contrast, the treatment with 18.5 °C remarkably decreased O_2_^.−^, H_2_O_2_ and MDA, and delayed the flowering and senescence. Furthermore, treatment with 18.5 °C significantly up-regulated the expression of glutamyl-tRNA reductase (Glu-TR) and magnesium chelatase (MgCH), and down-regulated the ferri chelatase (FeCH), protochlorophyllide oxidoreductase, chlorophyllase (CHLase), phaeophorbide a monooxygenase (PaO) and phytoene synthase (PSY), which further promoted the accumulation of chlorophyll (Chls) and reduced the carotenoids (Cars) in leaves. On the contrary, exposing to 28.5 °C remarkably down-regulated the Glu-TR and MgCH, and up-regulated the FeCH, CHLase, PaO and PSY, which in turn decreased the Chls and increased the Cars in tobacco leaves.

**Conclusion:**

As compared with the plants grown under 23.5 °C, lower (18.5 °C) and higher (28.5 °C) growth temperature inhibited the growth of tobacco plants. In general, treatment with 28.5 °C accelerated the flowering and senescence of tobacco plants by enhancing the accumulation of O_2_^.−^ and H_2_O_2_ in leaves, while exposing to 18.5 °C had the opposite effects. Treatment with 18.5 °C increased the content of Chls and reduced the Cars in leaves. In contrast, Treatment with 28.5 °C decreased the Chls and increased the Cars. Moreover, both O_2_^.−^ and H_2_O_2_ took part in the breakdown of Chls in tobacco leaves to some extent. The results suggest that growth temperature could regulate growth, development, and plastid pigments metabolism, and 23.5 °C could be an optimal temperature for growth, development and metabolism of plastid pigments of tobacco plants under the experimental conditions.

## Introduction

Environmental temperature is one of the key factors that affect the yield and quality of crop plants by influencing their distribution, major physiological and biochemical processes (Jumrani and Bhatia 2014; Pham et al. 2015; Souza Lucéia et al. 2016; Wang et al. 2016; Sunoj et al. 2016; Yang et al. 2016; Xu et al. 2016).

In nature, leaf senescence is a process of programmed cell death induced in age-dependent manner and by various environmental cues, such as senescence associated genes (SAGs), genotype, hormones, light and temperature, etc. (Yoshida 2003; Sarwat et al. 2013; Gao et al. 2016). Senescence is the final step in leaf development, which accompanied with the turn yellow of leaf, photosynthesis decline and biomacromolecules degradation, including nucleic acids, proteins, plastid pigments, and fatty acids, and so on. Finally, senescence is usually terminated with the death and abscission of leaf (Ougham et al. 2005; Lim et al. 2007; Zhang et al. 2011; Kim et al. 2016). Leaf is not only the important photosynthetic apparatus for plants but also the crucial edible part of many vegetables for human being. Both the pre-mature and late-mature of plants will greatly affect the yield and quality of many crop plants (Chéour et al. 1992; Navabpour et al. 2003; Kim et al. 2011).

Reactive oxygen species (ROS) can be continuously generated in plant cell as a kind of byproduct of aerobic metabolism (Møller and Sweetlove 2010). Generally, the production of ROS is maintained at lower levels. When the environment changes, such as temperature, go beyond the range required for normal growth and development of plants, excessive generation and accumulation of ROS will induce the oxidative stress, which in turn damage the cellular structures and many bioactive molecules, thereby accelerating the senescence and death of plants (Gill and Tuteja 2010; Suzuki et al. 2012). Previous studies suggested that the metabolism disturbance resulted in a rapid accumulation of ROS in cell is one of the predominant factors that inducing senescence of plants (Mccord and Fridovich 1969; Pastori and Rio 1994). Moreover, the peak of O_2_^.−^ generation was coincident with the initial of phase transformation of the membrane, which is caused by peroxidation of membrane lipids in senescing plant (Prochazkova and Wilhelmova 2007; Cheng et al. 2016). Additionally, it has been documented that both O_2_^.−^ and H_2_O_2_ take part in the destroy of chloroplast and the breakdown of plastid pigments (Brennan and Frenkel 1977; Adachi and Shimokawa 1995; Zhao et al. 2011).

Chlorophylls (Chls) and carotenoids (Cars) are the predominant plastid pigments in higher plants. Previous study has shown that Chls plays a central and indispensable role in the processes of photosynthetic light-harvesting and energy transduction of photosynthesis (Bollivar 2006). There are about 10^9^ tons of Chls biosynthesis per year, which closely associated with the growth, development, yield and quality of many crop plants (Moser et al. 2009). Therefore, the metabolism of Chls is considered to be one of the most important biochemical pathways on earth (Eckhardt et al. 2004).

In recent years, the synthesis and breakdown of Chls in higher plants have been extensively studied (Gossauer and Engel 1996; Hörtensteine and Kräutler 2011; Steccanella et al. 2015). At the first step of Chls biosynthesis, the formation of aminolevulinate acid (ALA) is the rate-controlling point of whole pathway. Thereby, glutamyl-tRNA reductase (Glu-TR) is the key enzyme for metabolic and environmental control (Kumar and Soll 2000; Gough et al. 2003). At the branch point of Chls biosynthetic pathway, magnesium chelatase (MgCH) and ferri chelatase (FeCH) compete for protoporphyrin (Proto) to synthesis of heme and Mg-Proto, respectively, which also is an important regulating step in Chls biosynthesis. In addition, the higher level accumulation of heme can lead to inhibition of the synthesis of Chls in plants by feedback regulation (Guo et al. 1998; Terry and Kendrick 1999; Brumann et al. 2014). At the later steps of Chls biosynthesis, the light-dependent protochlorophyllide oxidoreductase (POR) catalyzes the reduction of protochlorophyllide (Pchlide) to chlorophyllide (Chlide). In the processes, the tripolymer of the POR-NADPH-Pchlide was disaggregated, and the Chlide was released accompany with the breakdown of POR (Apel et al. 1980; Armstrong et al. 1995; Holtorf and Apel 1996; Reinbothe et al. 2010; Kim and Apel 2012; Talaat 2013; Menon et al. 2016). Finally, Chlide was catalyzed by chlorophyll synthetase (CHL) and chlorophyllide a oxygenase (CAO), respectively, and then turned into Chl a and Chl b, respectively (Beale 1999, 2005).

The Chls breakdown is an important process that influences the quality of many crop plants and agricultural products, during the leaf senescence. In the initial steps of Chls breakdown, Chl b is converted into *Chl a* through the Chls cycle. First, the phytol chain was cleaved from Chls molecular, which was catalyzed by chlorophyllase (CHLase) and generated chlorophyllide a (Chlide a). Secondly, the central Mg^2+^ was removed from Chlide by Mg-dechelatase (MDCase), and pheophorbide was subsequently catalyzed by phaeophorbide a monooxygenase (PaO) and turned into Red Chls catabolite (RCC). Finally, RCC was transformed into non-flourescent Chls catabolite (NCC). This whole process of Chls breakdown was usually named as ‘PaO pathway’ (Matile and Hörtensteinera 1999; Hörtensteiner 2006; Barry et al. 2008). Previous studies also have demonstrated that Chls catabolism is remarkably regulated by CHLase, MDCase and PaO in plants (Tanaka and Tanaka 2006; Ougham et al. 2008).

Carotenoids (Cars) are the second most abundant naturally occurring pigments on earth, with more than 750 members, which participate in various biological processes in plants, including photosynthesis, photoprotection, oxidation resistance, growth and development (Demming-Adams 1990; Bartley and Scolnik 1995; Stahl and Sies 2005; Shumskaya and Wurtzel 2013; Dias et al. 2014; Esteban et al. 2015; Liu et al. 2015). The first committed step in the process of Cars biosynthesis is the condensation of two geranylgeranyl diphosphate (GGPP) molecules to produce 15-cis phytoene, which is catalyzed by phytoene synthase (PSY) (Liu et al. 2015; Nisar et al. 2015).

Tobacco is one of the most important cash crop plants that widely cultivated worldwide. Since tobacco plants are a thermophilic crop plant originating from tropical region, they are sensitive to the change of temperature (Yamori et al. 2010; Popov et al. 2013; Zhang et al. 2013). It had been reported that the composition and contents of Chls and Cars in tobacco leaves are closely related to the quality and flavor of tobacco products (Kaneko and Harada 1972; Shigenage et al. 1994). Additionally, global temperature is predicted to increased 1.5–4.5 °C by the end of 21st century, and the growth, development, yield and quality of many crop plants will be negatively impacted by both sub- or sup-optimal temperatures (Porter 2005; Sunoj et al. 2016).

Numerous studies had been done to investigate the effects of different temperatures on plants growth and contents of Chls and Cars. However, in these studies, plants were usually exposed to cold or heat stress environment, and these researches were usually conducted under field condition. They were vulnerable to effect of variation of surrounding environment, such as light intensity, rainfall, humidity and temperature. Moreover, only the effects of the short-term or constant temperature on growth and Chls or Cars contents of plants had been examined (Tewari and Tripathy 1998; Zhao et al. 2007, 2011; Liu et al. 2012; Kalisz et al. 2016; Zhou et al. 2016). Until now, it is still unclear about the effects of long-term and non-stress temperatures on growth, development, senescence, and plastid pigments metabolism of plants. Therefore, the present study was conducted in climate chambers to evaluate the long-term effects of different and non-stress growth temperatures on growth, development and plastid pigments metabolism of tobacco plants.

## Materials and methods

### Plant material and experimental design

**Fig. 1:**
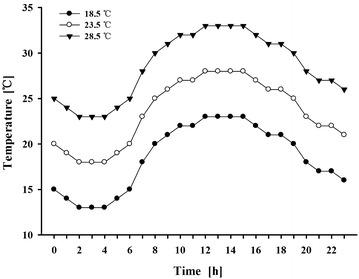
Different growth temperature settings in experiments

The experiment was conducted in climate chambers at Yunnan Academy of Tobacco Agricultural Sciences (24°34′N, 102°54′E) from March to August, during 2015 and 2016. Tobacco seeds (*Nicotiana tabacum* L. cv. Yunyan No. 87) were germinated in trays filled with a mixture of peat and vermiculite (2:1), and seedlings were grown under natural light in a greenhouse. The seedlings were transplanted into plastic buckets (volume: 3.14 × 0.04 × 0.5 m^3^) after 30 days. One seedling per bucket, and cultivated under field condition, till the 12th leaf expanded and leaf length reached 17 cm (marked as 0 day). These plants were then transferred into the climate chambers for the following cultivation under different growth temperatures (Fig. 1).

Three climate chambers (*Kulan Technology Co Ltd., Beijing, China*) were used in the study. Each chamber contained six lamp-supporting brackets with six high pressure sodium lamps (HPSL). Plants were grown under same light intensity (PPFD: 350 μmol m^−2^ s^−1^), relative humidity at 65%/50% (day/night), CO_2_ concentration at 400 ppm, and photoperiod 14 h/10 h (day: 06:00–19:59, night: 20:00–05:59). Eighty plants were grown in each chamber set to different temperature regimes of 18.5, 23.5 and 28.5 °C (daily average). Sampling of 12th leaf was carried out at 0, 15, 30, 45 and 60 days, and 12 plants were used each time, respectively, after exposed to different growth temperatures.

### Measurement of leaf length and leaf width

Length and width of 12th leaves were measured with a ruler.

### Assay of MDA and soluble protein content in tobacco leaves

MDA was extracted by 10% trichloroacetic acid (TCA), and mixed with 0.6% thiobarbituric acid (TBA). The MDA content was determined by UV/VIS spectrophotometer at 450, 532 and 600 nm, and expressed as nmol g^−1^ FW (Heath and Packer 1968).

Soluble protein content was assayed by coomassie brilliant blue method and determined at 595 nm by spectrophotometry, and expressed as mg g^−1^ FW (Braford 1976).

### Analysis of superoxide and hydrogen peroxide in tobacco leaves

Superoxide was extracted by Tris–Hcl buffer (50 mmol, pH 7.5) containing 1% polyvinylpyrrolidone (PVP) and EDTA (1 mmol L^−1^). The supernatant was mixed with Tris–Hcl buffer (50 mmol, pH 7.5) and 300 μL XTT (100 μmol L^−1^). The superoxide was analyzed by UV/VIS spectrophotometer (DU800, Beckman, USA) at 470 nm, and expressed as nmol g^−1^ FW (Frahy and Schopfer 2001).

Hydrogen peroxide was extracted by phosphate buffer (50 mM, pH 6.5) containing 1 mM hydroxylamine. And the extracted solution was mixed with 0.1% titanium sulfate in 20% (v/v) H_2_SO_4_. The H_2_O_2_ content was determined by UV/VIS spectrophotometer at 410 nm, and expressed as μmol g^−1^ FW (Li et al. 2012).

### Determination of Chls and Cars contents in tobacco leaves

Chls and Cars were extracted by 80% cold acetone, and determined at 663 (Chl a), 646 (Chl b) and 470 nm (Car) by UV/VIS spectrophotometer, and expressed as mg g^−1^ FW (Dere et al. 1998).

### Assay of relative mRNA abundance of genes by real-time PCR

Fresh 12th leaf tissue was sampled at 0, 15, 30, 45 and 60 days, respectively, and reserved in liquid nitrogen. 0.1 g leaf tissue was used for extraction of total RNA according to the instruction of RNAiso plus reagent (TakaRa, Japan). Total RNA was dissolved with RNase free H_2_O, and the cDNA was synthesis by PrimeScript RT reagent Kit with gDNA Eraser (Perfect Real Time,Takara, Japan). Sequence of genes, which were responsible for the biosynthesis and degradation of Chls and Cars was derived from the result of transcriptome analysis of our research team. The expression of genes in Chls and Cars metabolism pathway was detected according the method of SYBR Premix Ex Taq (Perfect Real Time, Takara, Japan). Primers for qPCR analysis were showed in Table 1.

**Table 1:** Primer sequences used in real-time PCR analysis

Gene and abbreviation	Forward primer (5′–3′)	Reverse primer
β-Actin	CTGAGGTCCTTTTCCAACCA	TACCCGGGAACATGGTAGAG
CL10247 Glutamyl-tRNA reductase (Glu-TR)	TGGTGTCCGTTTCGCTGT	GCTGTGGTATCTTCTGGCTTTTC
CL941. Contig 3 Ferri chelatase (FeCH)	GGACGAGAAATGAATCCTACCG	GCCCCGACAGAACAAAACA
Gi2318136 Magnesium chelatase (MgCH)	TGATGCCACATTCCAGAACC	GGCTTCTTCCCGTCTTTCCT
CL1870. Contig 2 Protochlorophyllide oxidoreductase (POR)	CCACGAAGAAACTGGCATTACA	CTGGAATGGAGGGAAAAGGAG
CL4058. Contig 1 Chlorophyllase (CHLase)	CTCTCTTCCCAGCTTGTGCTC	CCTTGCCTCTAATCCCTTTTGTT
Unigene40510_Rwg20A Mg-dechelatase (MDcase)	TCTATCCATCATCTGGCGTTTG	GGCTTCTTCCCGTCTTTCCT
Unigene38644_Rwg20A Phaeophorbide a monooxygenase (PaO)	ATCGCTTCGTCTTGGCATTT	TCTATCCATCATCTGGCGTTTG
CL4383.Contig 1 Phytoene synthase (PSY)	GGAATTTGGGCTTGTTGAGTG	TTGCGGAGTTATGTGGGATG

### Statistical analysis

All statistical analyses were conducted using *SPSS 11.5* (NY, USA) with the *Turkey’s text* (*P* < *0.05*), and the means and standard deviation (SD) were performed by analysis of variance (ANOVA) procedure using multiple comparisons. The figures were drawn with *Sigmaplot* 12.0 (*Systa software inc*., Chicago, IL, USA).

## Results

### Leaf length and width

**Fig. 2:**
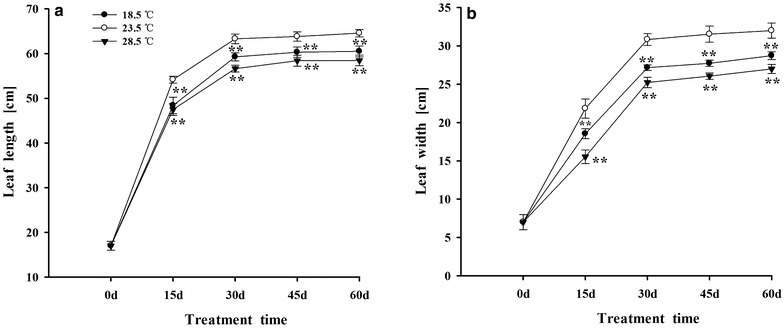
Effects of different growth temperatures on leaf length (**a**) and width (**b**) of tobacco leaves. Level of significant difference: ** and * indicate significant difference at P ≤ 0.01 and P ≤ 0.05, respectively, same as the following figures

The length and width of tobacco leaves were rapidly increased during 0–30 days, and reached the maximum size from 30 to 60 days. Treatment with 18.5 and 28.5 °C decreased their length and width of leaves, compared with plants under 23.5 °C (Fig. 2a, b).

### Contents of MDA and soluble protein in tobacco leaves

**Fig. 3:**
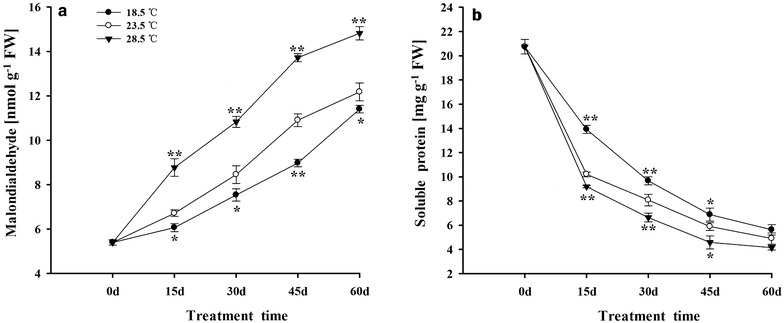
Effects of different growth temperatures on contents of MDA (**a**) and soluble protein (**b**) in tobacco leaves

The content of MDA was increased, while the soluble protein was decreased in tobacco leaves during 0–60 days. Compared with plants grown under 23.5 °C, treatment with 28.5 °C induced the accumulation of MDA in leaves, while exposing to 18.5 °C reduced the MDA content (Fig. 3a). Moreover, the reduction of the soluble protein in leaves was accelerated under 28.5 °C. On the contrary, the breakdown of soluble protein was slowed down under 18.5 °C, compared with plants grown under 23.5 °C (Fig. 3b).

### Levels of O_2_^.−^ and H_2_O_2_ in tobacco leaves

**Fig. 4:**
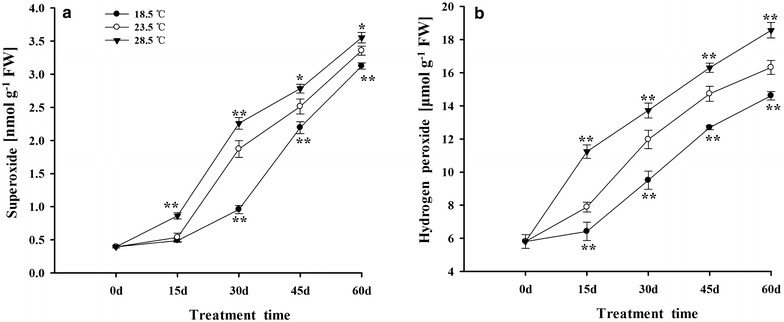
Effects of different growth temperatures on levels of superoxide (**a**) and hydrogen peroxide (**b**) in tobacco leaves

The levels of O_2_^.−^ and H_2_O_2_ were gradually increased in tobacco leaves during 0–60 days. Compared with plants grown under 23.5 °C, treatment with 28.5 °C enhanced the accumulation of O_2_^.−^ and H_2_O_2_ in leaves, while treatment with 18.5 °C decreased O_2_^.−^ and H_2_O_2_ levels (Fig. 4a, b).

### Accumulation of Chls in tobacco leaves

**Fig. 5:**
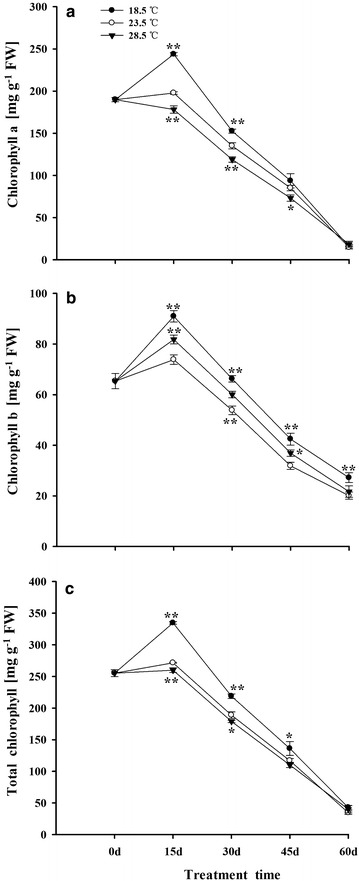
Effects of different growth temperatures on contents of Chla (**a**), Chlb (**b**) and total Chl (**c**) in tobacco leaves

The contents of Chla, Chlb and total Chls were gradually increased in tobacco leaves during 0–15 days, and then declined from 15 to 60 days (Fig. 5a–c). Compared with plants grown under 23.5 °C, the content of Chla, Chlb and total Chls in leaves increased under 18.5 °C, while the Chla and total Chls were decreased under 28.5 °C.

### Expression of Chls biosynthesis related genes in tobacco leaves

**Fig. 6:**
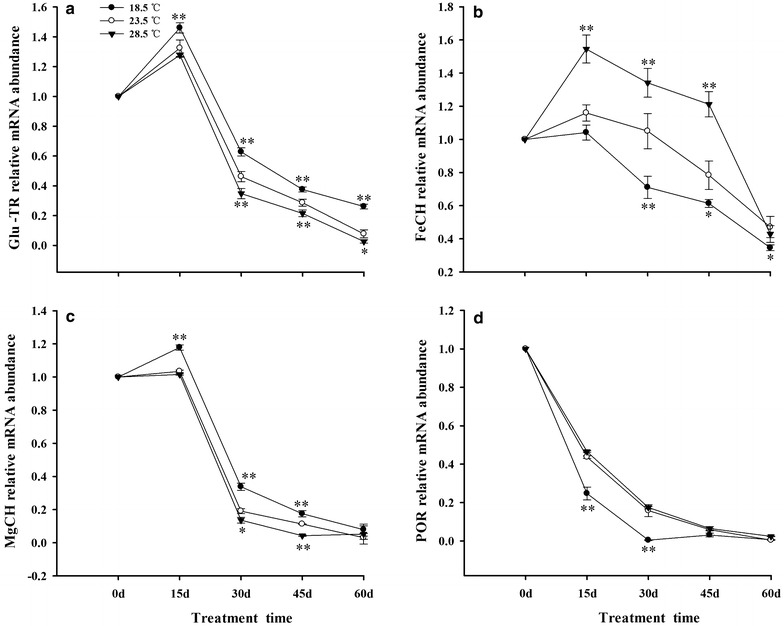
Effects of different growth temperatures on expression of Chls biosynthesis related-genes in tobacco leaves. Glu-TR (**a**); FeCH (**b**); MgCH (**c**); and POR (**d**), respectively

The expression of Glu-TR and MgCH were increased in tobacco leaves from 0 to 15 days, and then they decreased during 15–60 days. Compared with plants under 23.5 °C, Treatment with 18.5 °C up-regulated the expression of Glu-TR and MgCH but decreased the expression of POR and FeCH in leaves. Treatment with 28.5 °C down-regulated the expression of Glu-TR and MgCH but increased the expression of FeCH (Fig. 6).

### Expression of Chls degradation related-genes in tobacco leaves

**Fig. 7:**
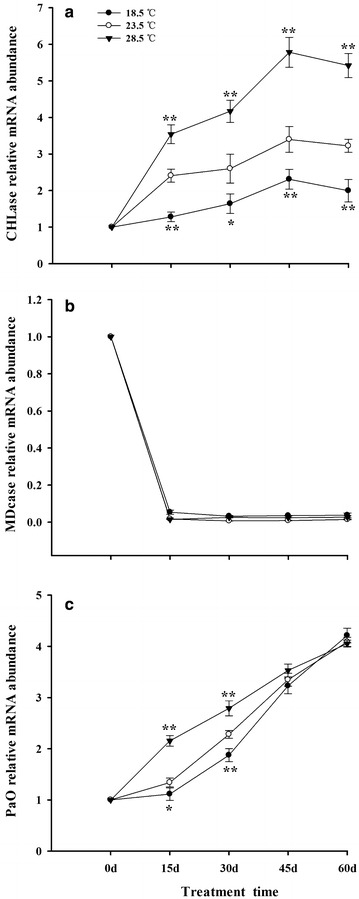
Effects of different growth temperatures on expression of Chls degradation related-genes in tobacco leaves. CHLase (**a**); MDcase (**b**); and PaO (**c**), respectively

In general, the expression of CHLase and PaO increased, while the MDCase was decreased in tobacco leaves during 0–60 days. Treatment with 28.5 °C significantly up-regulated the expression of CHLase and PaO in leaves. On the contrary, treatment with 18.5 °C down-regulated the expression of CHLase and PaO, compared with plants grown under 23.5 °C (Fig. 7a, c).

### Content of Cars and expression of PSY gene in tobacco leaves

**Fig. 8:**
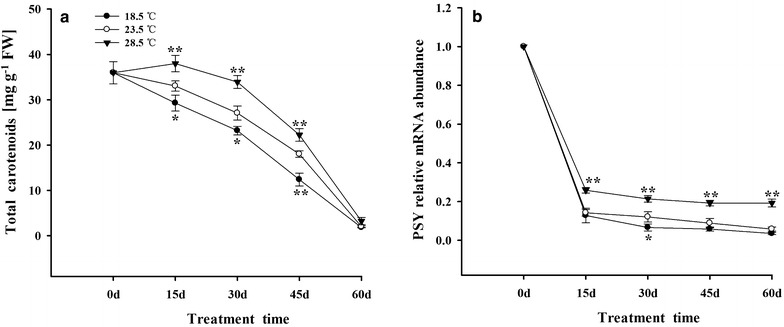
Effects of different growth temperatures on content of Cars (**a**) and expression of PSY gene (**b**) in tobacco leaves

The content of Cars and the expression of PSY gene in tobacco leaves were gradually decreased from 0 to 60 days. Compared with plants grown under 23.5 °C, the content of Cars and the expression of PSY gene increased when grown under 28.5 °C, and decreased under 18.5 °C (Fig. 8).

## Discussion

### Temperature affected the growth and development of tobacco plants

Temperature influences the distribution, growth, development and metabolism of plants (Sunoj et al. 2016; Yang et al. 2016; Xu et al. 2016). Our previous study also found that treatment with 16.5 and 30.5 °C remarkably inhibited the growth and photosynthesis of tobacco plants, compared with plants under 23.5 °C (Yang et al. 2017). In the present work, it was observed that tobacco plants showed distinct growth responses to different temperatures. Compared with plants grown under 23.5 °C, treatment with 18.5 and 28.5 °C significantly inhibited the growth and expansion of tobacco leaves (Fig. 2a, b). It was shown that both the relative lower temperature (16–18 °C) and the relative higher temperature (35 °C) remarkably inhibited the growth of leaves and reduced the leaf area of tomato, cherry and chicory plants, compared with the plants grown under medium ones (22–25 °C) (Venema et al. 1999; Mathieu et al. 2014). Plant morphogenesis was regulated by various environmental factors including light, temperature and so on (Berleth and Sachs 2001; Frankin 2009). In the present study, it was also observed that different temperatures significantly affected the morphogenesis of tobacco plants. Exposing to 18.5 °C promoted the elongation of petiole and stem, reduced leaf area and increased the angle between leaf and stem of tobacco plants. Whereas, treatment with 28.5 °C reduced plant height, leaf area, and the angle between leaf and stem of plants, compared with plants grown under 23.5 °C. Similar finding has also been observed when *Arabidopsis thaliana* plants were grown at 16, 22 and 28 °C (Frankin 2009).

Flowering was controlled by plant genotype, hormone and environmental factors (Cerdán and Chory 2003; Shalit et al. 2009; Song et al. 2013). This study observed that treatment with 28.5 °C significantly accelerated the flowering and senescence of tobacco plants; in contrast, treatment with 18.5 °C greatly delayed the flowering and senescence, as compared with those plants grown under 23.5 °C. Other study also demonstrated that 16 °C delayed the flowering time of *Arabidopsis thaliana* than those under 23 °C (Blázquez et al. 2003). Additionally, treatment of witloof plants with 35/28 °C accelerated the flowering than those plants under 17 °C (Mathieu et al. 2014).

### Temperature regulated the senescence of tobacco plants

Excessive accumulation of ROS in cell is one of the predominant factors inducing the senescence of plants. Moreover, the increase in MDA and the decrease in soluble protein in plants were normally considered the indicator of senescence of plants (Pastori and Rio 1994; Cheng et al. 2016). It was shown that the activities of proteolytic enzymes and the accumulation of ROS are increased during senescence of plants, accompanying with the rapid breakdown of proteins, especially the soluble proteins, and the increase in MDA in plants (Belknap and Garbarino 1996; Yoshida 2003; Lim et al. 2007). In the present study, treatment with 28.5 °C remarkably speeded up the degradation of soluble protein and increased the content of MDA in tobacco leaves, which in turn accelerated the senescence of tobacco plants. On the contrary, exposing to 18.5 °C slowed down the breakdown of soluble protein and reduced the content of MDA in leaves, followed by delaying of senescence of tobacco plants, when compared with plants grown under 23.5 °C (Fig. 3a, b). The finding had also been observed when wheat, sunflower and cucumber plants were grown at relative higher temperature of 33–44 °C (Ferguson et al. 1993; Zhao et al. 2011; Haba et al. 2014). Similarly, treatment with 15/13 °C (day/night) delayed the senescence of leaves in soybean, when compared with those under 30/25 °C (Kao 1980).

In the present work, the level of ROS was gradually increased in tobacco leaves during 0–60 days. In addition, treatment with 28.5 °C remarkably increased the levels of O_2_^.−^ and H_2_O_2_ in tobacco leaves, which in turn accelerated the process of senescence of tobacco plants. On the contrary, exposing to 18.5 °C significantly decreased the O_2_^.−^ and H_2_O_2_ levels in leaves, followed by delaying the senescence of tobacco leaves, when compared with plants grown under 23.5 °C (Fig. 4a, b). The above-mentioned results could be ascribed to the primary factors affecting senescence of tobacco plants, which speeded up by 28.5 °C, while delayed by 18.5 °C in the study. It has also been reported that high temperatures decreased the photosynthesis and increased the contents of O_2_^−^ and H_2_O_2_ in citrus, reed and tomato plants (Guo et al. 2006; Song et al. 2008; Ogweno et al. 2009). Additionally, it was demonstrated that higher temperature environment enhanced the generation and accumulation of ethylene, and then accelerated the senescence of soybean plants (Djanaguiraman and Prasad 2010), which can also be considered as a potential factor accelerating senescence of tobacco plants by 28.5 °C in this study.

### Temperature controlled metabolism of Chls in tobacco leaves

It had been shown that contents of Chls in plants are influenced by environmental temperature. It was observed that 18.5 °C significantly increased the content of total Chls in tobacco leaves, whereas exposing to 28.5 °C decreased Chls, when compared with plants grown under 23.5 °C (Fig. 5). Zhao et al. (2011) reported that 36 °C reduced the content of Chls in cucumber leaves. On the other hand, it was found that treatment with 16/14 °C (day/night) increased the level of Chls in lycopersicon leaves (Venema et al. 1999). It was also observed that the degradation rate of Chls is more rapidly than that of Cars in tobacco leaves, and this finding was also reported in other study (Whitfield and Rowan 1974). Additionally, Grover et al. (1986) have demonstrated that the senescence-induced degradation of Chls was faster at 35 °C than that at 25 °C in detached leaves of wheat, and Cars degradation was faster at 25 °C than that at 35 °C.

The accumulation of plastid pigments in plants is determined by both the processes of their synthesis and degradation. Glu-TR catalyzes the synthesis of ALA, which is a key step of biosynthesis of Chls in plants (Cornah et al. 2003). It was found that 18.5 °C increased the expression of Glu-TR and MgCH, but it decreased FeCH in tobacco leaves (Fig. 6), which in turn promoted the synthesis of Chls in leaves (Fig. 5). On the contrary, treatment with 28.5 °C significantly reduced the expression of Glu-TR and MgCH, but increased FeCH (Fig. 6), followed by reducing the content of Chls in leaves (Fig. 5), as compared with the plants grown under 23.5 °C. In addition, it had been reported that the enzyme activity of Glu-TR was also affected by accumulation of heme, suggesting that high level of heme would inhibit the activity of Glu-TR by feedback regulation and reduce the synthesis of Chls in plants (Terry and Kendrick 1999; Armas-Ricard et al. 2011). The higher expression of FeCH in leaves also was observed in the study, and this could be ascribed to the reduction of Chls in tobacco leaves under 28.5 °C. At the later steps of Chls biosynthesis in plants, POR catalyzes the conversion of Pchlide into Chlide, accompanying with the degradation of POR and the synthesis of Chls (Beale 1999, 2005; Kim and Apel 2012; Talaat 2013; Menon et al. 2016). In the present work, it was observed that 18.5 °C significantly reduced the expression of POR gene in tobacco leaves during 15–30 days (Fig. 6d), indicating that 18.5 °C accelerated the degradation of POR and the release of Chlide, which in turn promoted the synthesis of Chls in leaves, when compared with plants grown under 23.5 °C.

CHLase is considered as the first enzyme in the pathway of Chls breakdown, which is intimately concerned with the stability and degradation of Chls molecules in the chloroplast (Moll et al. 1978; Matile et al. 1997; Todorov et al. 2003; Harpaz-Saad et al. 2007). Generally, the activity of CHLase is decreased progressively during the senescence of plants, indicating that Chls degradation was also regulated by other factors (Yamauchi and Watada 1991; Minguez-Mosquera and Gallardo-Guerrero 1996). Results showed that treatment with different temperatures increased the expression of CHLase gene in tobacco leaves from 0 to 45 days, then decreased the expression from 45 to 60 days (Fig. 7a). Such a phenomenon has also been observed in leaves of *Phaseolus vulgaris* (Fang et al. 1998).

It was demonstrated that the level of endogenous abscisic acid (ABA) was increased and the leaf senescence was accelerated during the process of growth and development of plant (Zhang et al. 2012). Moreover, the accumulation of ABA was elevated when plants were grown under stress conditions, such as drought, chilling and extreme temperature, followed by up-regulating the expression of CHLase of plants (Guo and Gan 2005; Gupta et al. 2012). Additionally, it was reported that high temperature enhanced the de novo synthesis of CHLase in plants (Trebitsh et al. 1993; Gong and Mattheis 2003). These could be considered to be the primary factors for the increase in expression of CHLase gene in tobacco leaves under 28.5 °C, which in turn reduced the content of Chls in leaves in this study.

Removal of Mg^2+^ from the Chlide is an important step in Chls breakdown pathway and leaf senescence (Costa et al. 2002). In the present work, expression of MDcase gene was drastically decreased in tobacco leaves from 0 to 15 days, then maintained at a lower level during 15–60 days. No significant difference of MDcase was observed in leaves among different growth temperature treatments (Fig. 7b). Previous study demonstrated that the stable activity of MDCase depends on the continuously synthesis of soluble protein of plants (Langmeier et al. 1993). However, it was observed that content of soluble protein was decreased during the process of senescence of tobacco leaves, which could be responsible for the decrease of expression of MDCase gene in tobacco leaves.

Finally, PaO converts the pheophorbide into RCC, which is induced by senescence of plants (Smart 1994; Hörtensteiner et al. 1995; Matile and Hörtensteinera 1999; Hörtensteiner 2006). In this work, treatment of plants with 28.5 °C up-regulated the expression of PaO gene, followed by accelerating the breakdown of Chls in tobacco leaves. On the contrary, exposing to 18.5 °C significantly down-regulated the PaO and slowed down the degradation of Chls in leaves, when compared with the plants grown under 23.5 °C (Fig. 7c). Previous study has also found that treatment with heat shock increased the activity of PaO, followed by reducing the content of Chls and accelerating the senescence of barley leaves (Rodoni et al. 1998). Our results suggest that treatment with 28.5 °C speeded up the breakdown of Chls by up-regulating the expression of CHLase and PaO genes in tobacco leaves, and exposing to 18.5 °C slowed down the degradation of Chls by down-regulating the expression of CHLase and PaO genes in the leaves.

Furthermore, the change in level of ROS was coincident with the variation of content of Chls in tobacco leaves under different growth temperatures (Figs. 4, 7), which indicated that ROS takes part in the breakdown of Chls in leaves to some extent. Similar results also had been reported in the previous studies (Brennan and Frenkel 1977; Adachi and Shimokawa 1995; Zhao et al. 2011).

### Temperature influenced metabolism of Cars in tobacco leaves

Previous studies showed that PSY is the key enzyme for synthesis of Cars in plants, and overexpression of PSY gene remarkably increased the content of Cars in *Brassica napus* (Shewmaker et al. 1999; Shumskaya and Wurtzel 2013). In the present work, it was observed that 28.5 °C significantly increased the expression of PSY gene in tobacco leaves, which in turn increased the accumulation of Cars in leaves, when compared with these plants grown under 23.5 and 18.5 °C (Fig. 8a, b). Lefsrud and Kopsell (2005) have reported that the content of Cars was the highest in kale under 30 °C than those at 15, 20 and 25 °C. Moreover, Cars content was increased linearly with increasing air temperature. Additionally, Ikoma et al. (2001) have also showed that the PSY transcript in mature leaves of Satsuma mandarin fruit was higher than in young ones. These could be ascribed to the increase in content of Cars in tobacco leaves under 28.5 °C in the present study.

## Conclusion

Different temperatures affected the growth, development and morphogenesis of tobacco plants. Compared with tobacco plants grown under 23.5 °C, treatment with 18.5 and 28.5 °C significantly inhibited the growth of tobacco plants. Treatment with 28.5 °C increased the contents of O_2_^.−^, H_2_O_2_, MDA and Cars and reduced the contents of soluble protein and Chls in tobacco leaves, followed by accelerating the flowering, maturity and senescence of tobacco plants. On the other hand, exposing to 18.5 °C significantly decreased the contents of O_2_^.−^, H_2_O_2_, MDA and Cars and increased the contents of soluble protein and Chls in tobacco leaves, which in turn slowed down the flowering, maturity and senescence, as compared with the plants grown under 23.5 °C. Moreover, both the O_2_^.−^ and H_2_O_2_ participated in the degradation of Chls in tobacco leaves to some extent. As a result temperature of 23.5 °C is more beneficial for the growth, development and metabolism of plastid pigments of tobacco plants under the experimental conditions of this study.

## Abbreviations

Chl: chlorophyll
Car: carotenoid
CHLase: chlorophyllase
FeCH: ferri chelatase
Glu-TR: glutamyl-tRNA reductase
H_2_O_2_: hydrogen peroxide
MDA: malondialdehyde
MDCase: Mg-dechelatase
MgCH: H subunit of magnesium chelatase
O_2_^.−^: superoxide
PaO: phaeophorbide a monooxygenase
POR: protochlorophyllide oxidoreductase
PSY: phytoene synthase
